# A technical solution to a professional problem: The risk management functions of prognosticators in the context of prognostication post-cardiac arrest

**DOI:** 10.3389/fsoc.2022.804573

**Published:** 2022-08-19

**Authors:** Sarah Elizabeth Field-Richards, Stephen Timmons

**Affiliations:** ^1^School of Health Sciences, University of Nottingham, Nottingham, United Kingdom; ^2^Nottingham University Business School, University of Nottingham, Nottingham, United Kingdom

**Keywords:** prognostication, prognosticator, cardiac arrest, risk, professions, risk management, risk work, sociology of prognosis

## Abstract

Cardiac arrest (CA) is a major cause of mortality and morbidity globally. Two-thirds of deaths among patients admitted to intensive care units following out-of-hospital CA are due to neurological injury, with most as a consequence of withdrawing life-sustaining treatment, following prognostication of unfavorable neurological outcome. Given the ramifications of prognosis for patient outcome, post-cardiac arrest (P-CA) guidelines stress the importance of minimizing the risk of falsely pessimistic predictions. Although prognosticator use is advocated to this end, 100% accurate prognosticators remain elusive, therefore prognostication P-CA remains pervaded by uncertainty and risk. Bioethical discourse notwithstanding, when located within a wider socio-cultural context, prognostication can be seen to present risk and uncertainty challenges of a professional nature. Such challenges do not, however, subvert the medical profession's moral and ethical prognostication obligation. We interpret prognosticator use as an attempt to manage *professional* risk presented by prognostication P-CA and demonstrate how through performing “risk work,” prognosticators serve professional functions, mediating tension between the professional duty to prognosticate, and risk presented. We draw on sociological analyses of risk and uncertainty, and the professions to explicate these (hitherto less enunciated) professional risk management functions of prognosticators. Accordingly, the use of prognosticators is conceived of as a professional response – a technical/scientific solution to the problem of professional risk, inherent within the P-CA prognostication process.

## Introduction

Cardiac arrest (CA) is a major cause of mortality and morbidity globally and outcomes for resuscitated patients are typically poor. Two-thirds of deaths amongst patients admitted to intensive care units following CA outside of hospital are due to neurological injury. Most of these deaths are a consequence of withdrawing life-sustaining treatment, following prognostication of an unfavorable neurological outcome - severe neurological injury, persistent vegetative state, or death (Nolan et al., 2015; Sandroni and Geocadin, 2015). Given the ramifications of prognosis for treatment decisions and patient outcome, post-cardiac arrest (P-CA) guidelines stress the importance of minimizing “the risk of a falsely pessimistic prediction” (Nolan et al., 2015). Prognostication P-CA is however challenging and inexact and the use of clinical indicators (prognosticators) is advocated to support medical professionals' decision-making (Horn et al., 2014; Sandroni and Geocadin, 2015). The various prognosticators employed by medical professionals can be classified as clinical examination (e.g., Glasgow Coma Scale score, corneal reflex, seizure presence), neurophysiological studies (e.g., electroencephalography, somatosensory evoked potentials), biochemical markers (e.g., neuron-specific enolase), and imaging studies (magnetic resonance imaging, brain computerized tomography) (Nolan et al., 2015).

The dominant biomedical narrative and rationale supporting prognosticator use P-CA is normatively framed in bioethical terms concerning patient “best interest,” avoiding inappropriate treatment “in patients with no chance of recovery,” whilst conversely avoiding prematurely withholding treatment for those with “a chance for good neurologic outcome” (Sandroni and Geocadin, 2015, p. 2). Further, bioethically, prognosticators should have a specificity of 100% or a false-positive rate of zero (i.e., when they predict a poor outcome, no patient should experience a long-term “good” outcome) (Nolan et al., 2015). However, despite an abundance of research (see Sandroni and Geocadin, 2015; Sandroni et al., 2018), definitive prognosticators remain elusive, with Nolan et al. (2015) concluding that none are adequate for the task asked of them. Though clinically varied, prognosticators can be considered equivalent as they are employed for a common purpose (prognostication) and one for which they are ultimately inadequate. As such, guidelines recommend a multi-modal approach involving multiple types of brain injury tests (Nolan et al., 2015), which might be interpreted as an attempt to triangulate prognosticator uncertainty. Although “Even the most robust predictors … do not guarantee an absolute certainty when predicting poor outcome” (Sandroni and Geocadin, 2015, p. 6), usage continues, creating tension between the bioethical imperative for prognostic certainty and the “probabilistic knowledge” (Gale et al., 2016, p. 1062) provided by uncertain prognosticators. Paradoxically, since 100% accurate prognosticators remain to be found, their use as a means of managing clinical and bioethical risk and uncertainty itself, constitutes an uncertain practice entailing the risk of error, reflecting tension between prognosticators as both creators and managers of risk and rendering the quest for certainty inherently uncertain.

### Origins and nature of the paper

This paper presents a theoretical interpretation of the professional functions served by a documented empirical phenomenon (prognostication P-CA). The stimulus for the paper was a review of scientific biomedical research pertaining to prognostication P-CA, conducted in 2013, involving 185 returned papers. Through the literature review process, the authors became familiar with scientific and biomedical aspects of the P-CA prognostication process, which served as a point of departure for sociological thinking. The biomedical literature suggested that definitive prognosticators remained to be identified since the publication of the 2010 Resuscitation Council (UK) guidelines (Deakin et al., 2010). The review also demonstrated a significant and sustained research focus and professional interest in the identification of accurate P-CA prognosticators. This focus on the identification of accurate P-CA prognosticators remains today (e.g., Sandrioni et al., 2020; Andersson et al., 2021; Oh et al., 2021), reflecting their apparent importance in managing clinical and bioethical risk and uncertainty associated with patient outcome P-CA, as an important and pressing medical problem.

The bioethical rationale, function and research notwithstanding, as sociologists of the professions considering phenomena within a wider socio-cultural context, we theorized as to how the quest for certainty might be conceived of, and the professional functions that prognostication P-CA serves and to what effect. We also observed a tension created by the professional duty to engage in the process of prognostication on the one hand, as a process pervaded by uncertainty and professional risk stemming from the use of uncertain prognosticators, on the other. We considered how this tension might be managed by medical professionals and the overall salience of the prognostication P-CA process for the medical profession. During this process, we engaged with sociological literature relating to the professions (particularly the medical profession, including areas such as medical prognosis, death and dying), and risk and uncertainty, in informing and developing our theorizing, applying existing sociological knowledge to a new, specific context (prognostication P-CA). Accordingly, references to this literature are included throughout the paper to support the arguments presented. Although a theoretical rather than empirical paper in nature, the arguments presented herein can therefore be considered to be empirically grounded in considering a documented empirical phenomenon (prognostication P-CA) observed in the biomedical literature, and in applying empirically-based sociological theory to analyze how it can function for professional means.

### Argument and structure of the paper

In this paper, we argue that prognosticator use P-CA can be interpreted as an attempt to manage not only clinical and bioethical risk but also *professional* risk presented by the prognostication process. Bioethical discourse notwithstanding, prognostication presents risk and uncertainty challenges of a *professional* nature. Such challenges do not however subvert the medical profession's moral and ethical prognostication obligation (Christakis, 1999). We demonstrate how although uncertain prognosticators can create risk, by negotiating professional challenges through performing “risk work” (Gale et al., 2016, p. 1046), prognosticators function to manage professional risk in multiple ways, with varying outcomes. In this way, we show how prognosticators serve professional functions, mediating tension between the professional duty to prognosticate on the one hand and the challenges it presents, in terms of professional risk and uncertainty, on the other. Their use constitutes a professional response - a technical/scientific solution to the professional problem of risk and uncertainty arising from the P-CA prognostication process. Whilst prognosticators have as yet failed to deliver certainty in relation to prognostication of patient outcome P-CA as their “primary” purpose, when considered within a wider socio-cultural context, their use can therefore be observed to serve other important professional risk and uncertainty management functions. In explicating our argument, we utilize extant theory and empirical work, applying and extending insights to an analysis of the P-CA prognostication context. In doing so, we contribute to knowledge and theorizing in the sociological areas of risk and uncertainty, and professions. Further, through a critique of the process and practice of prognostication more broadly, we contribute to the “emerging sociology of prognosis” (Timmermans and Strivers, 2018, p. 13).

In the sections that follow, firstly, we outline the theoretical positioning of the paper and locate the central argument within the perspectives introduced. We then identify professional risks presented by the P-CA prognostication process and the way prognosticators function to manage them, through various forms of risk work. We do this by presenting a series of inter-related arguments, each focusing on an area of professional risk and considering the nature of the risk work performed by prognosticators, how this functions to address the professional risk, and to what effect. Collectively, these arguments demonstrate the risk work role that prognosticators perform in managing professional risk presented by the prognostication process P-CA. Before concluding the paper, we summarize and provide a theoretical model of the arguments presented and discuss contributions to knowledge, caveats and opportunities for research.

## Theoretical approach

The theoretical position adopted in this paper is informed by the sociology of professions and the sociology of risk and uncertainty. These approaches are now outlined and contextualized in terms of their relevance to the central argument of the paper.

### Sociology of professions

Our paper considers the P-CA prognostication process within the specific professional context of medical professionals. Acknowledging different possible approaches to “profession” (Macdonald, 1995), we now outline our position. For us, “profession” is a socially constructed concept, serving as much to create a market shelter for an occupational group as it does to analytically delineate between types of work (Freidson, 1970). Within the sociology of professions, we align ourselves with the neo-Weberian but eclectic position of Saks (2016), which draws centrally on the work of authors including Larson (1977) (the professional project) and Abbott (1988) (the system of professions). Saks argues for the continued relevance of the neo-Weberian approach, despite the critique of, for instance, Evetts (2006). Professions are engaged in an ongoing professional project where they attempt to secure privileges (principally from the state), such as control over work and power to accredit members of their profession. The main strategy used by professionals to accomplish this is by defining a “jurisdiction” where they have an effective monopoly on professional knowledge, enabling control over work and limitation of interference from the state (and managers) (Abbott, 1988). Contemporary sociology of professions theory is dominated by questions around hybridity, where professionals also function as managers (Noordegraaf, 2015; Breit et al., 2017). As we shall see, medical professionals hold resource management responsibilities in P-CA contexts and can be seen as inhabiting these hybrid roles.

Informed by the sociology of professions, we focus on ways in which the P-CA prognostication process presents professional risks - to central tenets of professional status. We interpret the use of prognosticators to manage professional risk, as a form of professional defense mechanism - part of the medical profession's engagement in their ongoing professional project to gain and maintain professional status and power.

### Sociology of risk and uncertainty

We draw on the broad and diverse sociology of risk and uncertainty as our other key theoretical approach. Consistent with our approach to “profession”, we view risk as socially constructed. As it demonstrates “best fit” with the issues considered in this paper, focused as they are on medical professionals' practice of prognosticator use to manage risk, we employ the “risk work” approach of Gale et al. (2016). This approach “aims to make visible *working practices to assess or manage risk*, in order to subject these practices to sociological critique” (Gale et al., 2016, p. 1046). The risk work performed by prognosticators reflects all of Gale et al.'s (2016) aspects of risk work; translation of risk, minimizing risk, and caring in contexts of risk. In accordance with this approach, we consider prognosticator use itself to constitute a form of professional “risk work,” a working practice employed by physicians to assess and manage (professional) risk presented by the P-CA prognostication process. We subject this practice to sociological critique by explicating the various discrete risk work roles and functions that prognosticators play in managing professional risk - how prognosticators perform risk work on behalf of medical professionals and to what effect.

## The risk of “getting it wrong” – Managing risks to professional credibility, trust, power, and the role of scientific rationality

This section describes the role of prognosticators and their association with scientific rationality, in managing professional risks presented by the prognostication process, to professional credibility, trust and power. The specific mechanisms through which prognosticators perform the professional risk work characterizing this risk management are identified.

Prognostication is a central component of the medical profession's jurisdiction (Kellett, 2008). Consonant with knowledge and expertise forming part of the profession's social licensure and contract (Bhugra, 2014), there is a social expectation that prognostication will be accurate and consistent with outcome (Christakis, 1999, 2003). Competence in “getting it (prognosis) right” (an accurate prognosis) therefore plays an important role in maintaining professional credibility and trust afforded to the medical profession by society, and ultimately, in the maintenance of professional power and status (Cruess, 2006; Bhugra, 2014). However, the inexact and uncertain nature of prognostication P-CA in particular, can be seen to present the medical profession with an enhanced risk of “getting it (prognosis} wrong” (an inaccurate prognosis), resulting in the potential for inappropriate withdrawal of treatment, or conversely, prediction of a positive outcome where the actual outcome transpires as poor. Given the centrality of “getting it right” to notions of credibility, trust, and ultimately professional power, the risk of “getting it wrong” can in turn be interpreted as presenting a professional risk to these aspects of professional status - individually, amongst individual clinicians, or collectively, to the medical profession as a whole. These risks associated with “getting it wrong” are compounded by the high bioethical stakes associated with patient outcome in the P-CA context. Prognosticators can assist in managing these professional risks to credibility, trust and power, stemming from the risk of “getting it wrong,” in a two-fold way - by performing risk work involving the *mitigation* of risk, and the facilitation of a *devolving-dispersing-diluting-delegating* process. How prognosticators act to allow the management of professional risk through the performance of risk work will now be elaborated upon in turn.

### Managing professional risk through mitigation

“Prognosticators” in their various guises constitute an array of clinical data. When located and interpreted within the context of the wider P-CA scientific evidence base, however, the meaning and relevance of clinical data for P-CA outcomes become realized. “Clinical data” undergo a process of epistemological translation, becoming “prognostic markers” and allowing for the status of scientific “prognosticators” to be assigned. Considered in isolation from the wider evidence base, clinical data alone hold little relevance and utility for the prognostication process. Through the processes of interpretation and classification, however, prognosticators become imbued with powerful significance as “markers of meaning” and “scientific evidence” in the context of prognostication P-CA. In turn, once established, prognosticators act as technological (and epistemological) “keys,” unlocking and granting medical professionals access to the scientific knowledge necessary to inform prognostic decision-making. In interpreting individual patient data within the context of existing scientific knowledge, a (probable) prognosis is indicated in accordance with the scientific knowledge relevant to outcomes P-CA. In this way, the certainty surrounding prognosis is increased (“getting it right”), and the risk of “getting it wrong” is reduced. Reducing the risk of “getting it wrong” in turn reduces associated professional risks to credibility, trust and power, inherent in the prognostication process. As part of their risk work, through reducing the risk of “getting it wrong,” the use of prognosticators in conjunction with scientific knowledge (to which they facilitate access), therefore acts to manage the professional risk presented by the prognostication process, through the risk work mechanism of *mitigation*. Professional risks to credibility, trust and power are mitigated by reducing the risk of “getting it wrong.” Whilst reducing risk, owing to the technical indeterminacy of prognosticators, the risk of “getting it wrong” is not eliminated however and a prognosis, though legitimate, may be inaccurate.

### Managing professional risk through devolving-dispersing-diluting-delegating

The above notwithstanding, in situations where “getting it wrong” materializes [despite the use of (inherently uncertain) science], the use of prognosticators in conjunction with science can function to manage professional risk through risk work in this situation also. This is done through (partially) *devolving* decision-making, *dispersing* attribution for “error,” and *diluting* and *delegating* the locus of responsibility, and professional risk, as a result.

The use of prognosticators, coupled with their scientific foundation, provides professionals with a clinical rationale that guides and underpins their prognostic decision-making, and which can be appealed to as justification, in instances where a professional might “get it wrong.” When adopting “evidence-based” decision-making, the individual decision-making becomes situated within, informed by, and is therefore partially attributable to, a wider knowledge community. “Scientific” decision-making, facilitated by prognosticator use, could therefore be interpreted as a form of partially devolved decision-making. In instances where an individual “gets it wrong,” attribution of “error” might be seen to be less concentrated on the “incorrect” prognostic decision-making of one clinician and instead be more dispersed and distributed amongst the more nebulous knowledge network. “Getting it wrong” is then at least partially attributable to the collective conglomerate of the scientific community and knowledge, to which the individuals' decision-making was (at least partly) influenced and devolved. In turn, the locus of responsibility for “getting it wrong” might also be seen to be more diffuse and dilute, in terms of its concentration and attribution to individual professionals and professions, and partly relocated and delegated to prognosticators and science. In the process of delegating responsibility for "getting it wrong", so too are associated professional risks to credibility, trust, and power. As such, in instances where individuals “get it wrong,” the use of prognosticators in conjunction with “science” functions to manage professional risk via risk work. This risk work involves the facilitation of *devolved* prognostic decision-making as a means of *dispersing* attribution for “error,” *diluting* the professional locus of responsibility, and *delegating* it to prognosticators and science, and with it, professional risk (to credibility, trust and power) associated with “getting it wrong”.

### The role of scientific rationality and dirty work

The mechanism by which the risk work of prognosticators can function to manage professional risk by devolving-dispersing-diluting-delegating, is illuminated by considering the nature and status of prognosticators and the evidence base, as *scientific* forms of knowledge and proxies for *scientific* rationality. As a socially and professionally trusted, hegemonic form of knowledge (Aronowitz, 1988), the “scientific” affinity of prognosticators affords them the power to function in these ways to manage professional risk. Since the clinical rationale guiding and underpinning a prognosis P-CA is derived from *scientific* evidence (a professionally endorsed, socially legitimate resource), when “getting it wrong,” this can be (at least partly) attributed to a “fault” inherent within the scientific prognostication process itself (the inexact science of prognosticators). As such, responsibility can be (partially) located within the scientific, rather than the professional realm, allowing for professional credibility, trust and power to be maintained. The scientific status of prognosticators, therefore, renders them both the means (a facilitator) and the object of professional risk delegation.

The risk work of prognosticators, in relation to the delegation of responsibility and professional risk, might be conceived of as a form of “boundary work” (Gieryn, 1983; Fournier, 2000), serving to delegate “dirty work” (Hughes, 1958) externally from the professional, to the scientific realm. Traditionally, “boundary work” is used within the sociology of professions to refer to the negotiation of inter- or intra-professional boundaries by the professions. In facilitating the delegation of responsibility and risk from the professional to the scientific realm, the risk work performed by prognosticators in the P-CA context might be considered as an enactment of boundary work by prognosticators *on behalf of* medical professionals - or boundary work by proxy. In addition, the uncertain, risky, and clinically, bioethically and professionally challenging nature of prognostication P-CA might allow the prognostication process to be conceived of as “dirty work.” In delegating responsibility and risk associated with prognostication to the scientific realm, we argue that boundary work performed as part of the prognosticators' risk work role, functions to delegate professional dirty work. This delegation of dirty work constitutes a further affordance of the risk work role played by prognosticators in managing professional risk presented by the P-CA prognostication process. Medical professionals have been documented to use boundary work discursively as a means of removing their practice from legally and ethically contentious issues (dirty work) in other contexts (Miner, 2019). Here, with prognosticators constituting both the means and object of risk delegation, we show that boundary work can be both performed by prognosticators *on behalf of* medical professionals (boundary work *by proxy*), which *also* serves to delegate dirty work to prognosticators and science.

## The risk of death, “getting it right” and the (unwanted) power of prophecy – Managing risks to professional power, control and identity

This section describes the role of prognosticators in managing risks to professional power, control and identity, presented by the prognostication process. The place of calculability, predictability and prophecy within this risk work is considered.

Patients' P-CA can be conceived of as occupying an uncertain, liminal space “‘betwixt and between' living and dying” (Nicholson et al., 2012, p. 1426). In this context, prognostication presents a “particularly thorny form of uncertainty” since paradoxically it constitutes a medical situation with high levels of unpredictability, which both demands and subverts professional efforts to prognose (Christakis, 2003, p. 140). Further, where it includes the possibility of death, prognostication entails physicians considering and confronting uncertainty and risk in relation to patient (and personal) mortality (Christakis, 1999). With its high degree of uncertainty and high risk of death, together with heightened bioethical consequences, this is especially true of and intensified within the context of prognostication P-CA. To physicians, death can be considered a professionally “noxious and worrisome” stimulus, which impinges upon their social role (Christakis, 2003, p. 135). Since “cure” occupies a central place in the medical professional role and identity, death (as indicative of the absence or futility of cure) can be viewed as connoting professional failure, presenting challenges to this professional role and identity (Christakis, 2003; Apesoa-Varano et al., 2011). In addition, death serves as a stark reminder of the limits of medical knowledge, control and power, and as such, presents risks to professional power and control. Through association with death, the prognostication process can therefore be seen to confront professional power, control, and identity which are compounded and heightened by the high risk and highly uncertain nature of P-CA in relation to patient mortality. Although inherent within medical professionals' social role, through association with death, prognostication also presents challenges to it, which Christakis (2003, p. 140) suggests creates “sociological anxiety” amongst physicians. We argue that the use of prognosticators by medical professionals in the context of prognostication P-CA can function to manage professional risks presented indirectly, through introducing (a sense of) calculability and predictability surrounding death, and more directly (and less desirably) through the action of a self-fulfilling prophecy.

### Indirect management of professional risks – The role of calculability and predictability

Section “The risk of “getting it wrong” – Managing risks to professional credibility, trust, power and the role of scientific rationality” identified that the use of prognosticators and wider scientific knowledge in the prognostication process is intended to increase certainty surrounding prognosis (“getting it right”), thereby reducing the risk of medical professionals “getting it wrong.” Further, we argue that by increasing certainty surrounding prognosis, the use of prognosticators serves to introduce calculability and predictability surrounding death (both actual and/or a sense of) thereby rendering death more calculable. In cases where a professional ultimately “gets it right,” prognosticators might be considered to have introduced both actual, and a sense of, calculability and predictability. Where patient outcome is consistent with prognosis, the calculability and predictability provided by prognosticators transpire and are confirmed “in actuality.” In cases where a professional “gets it wrong,” however, *a sense of* calculability and predictability surrounding outcomes is nonetheless introduced, despite the absence of calculability and predictability being realized “actually.” Prognosticator use *in itself* confers a subjective sense of calculability and predictability regarding death, regardless of the objective accuracy of predictive capabilities of prognosticators (and the “actual” degree of calculability and predictability they provide) in relation to patient outcome. This sense of calculability and predictability is as significant as objective accuracy, in terms of the function of prognosticators in managing professional risk concerning death.

The act of prognostication alone does not allow medical professionals to (re)gain “power” over death directly (patient outcome remains the same). The (sense of) calculability and predictability introduced by prognosticators does however afford the (re)assertion and (re)establishment of medical power and control over death - either “felt” or more materially, by way of allowing for the initiation of palliative care and psychosocial preparatory rituals, for example. In these instances, although medical professionals do not directly influence *whether* patients die, they can influence *how* they die. Drawing on risk theory, which suggests that when risk becomes calculable, it becomes manageable (Streicher et al., 2018), in rendering death more calculable, it becomes more predictable, manageable and tolerable. Similarly, the calculability introduced by prognosticators might be interpreted as a means of “scientizing” death, medicalizing the uncertain and liminal space between living and dying, and increasing professionals' (sense of) controllability. In affording medical professionals (a sense of) calculability and predictability, and in turn (a perception of) control over death, through their risk work, the use of prognosticators functions to allay professional risks to power, control and identity stemming from the confrontation with death, presented by the P-CA prognostication process.

### Direct management of professional risks – (unwanted) professional power and the self-fulfilling prophecy

We have argued that the act of prognostication alone does not change patient outcome and therefore medical professionals do not (re)gain power over death directly through the act of prognostication. We acknowledge however that in the case of prognostication P-CA particularly, a prognosis of death (futility) can lead to subsequent withdrawal of artificial life-sustaining treatment. This likely leads to the outcome of death predicted, therefore becoming a self-fulfilling prophecy (Christakis, 1999; Nolan et al., 2015); the prophecy of death becomes fulfilled by the course of action warranted by the prognosis of futility. In these instances, prognosticators have more direct implications for professional power concerning death – their use allows medical professionals to (re)gain power and control over death more directly and concretely. Although within the sociology of professions, power is commonly analyzed in a professionally desirable way, this is an instance where power and its exercise are more professionally problematic and somewhat less palatable. The risk work performed by prognosticators in relation to managing professional risks to power, control and identity, associated with confronting death as part of the prognostication process, can be seen, therefore, to serve in two ways. It can serve indirectly, through instilling (a sense of) predictability, calculability, and thus power and control over death indirectly, and more directly (and uncomfortably), by affording professionals more direct power and control over death, through the mechanism of a self-fulfilling prophecy.

## The risk of managerialism and new professionalism – Managing risks to professional norms, integrity, and identity

This section describes the protective and mediating role of prognosticators in managing risks to professional fundamental norms, integrity and identity, presented by the P-CA prognostication process, when considered in the context of managerialism.

Medical professionals are becoming increasingly managerialized in the context of contemporary healthcare (Numerato et al., 2012). Physicians act as front-line “gatekeepers” to finite healthcare resources (Kluge, 2007, p. 57) and have been tasked with and responsibilized for resource management, symptomatic of a “‘new' professionalism” accompanying the managerialist milieu (Evetts, 2011, p. 406). Managerially-driven resource allocation responsibilities, however, present professional and ethical challenges (Christakis, 1999; Kluge, 2007; Mechanic, 2008). This is especially true in instances where managerial responsibilities might come into conflict with a patient's interest. In medical ethics literature, this is referred to as “bedside rationing” (e.g., see Ubel and Goold, 1997).

Medical intervention is a costly and finite resource. A prognosis of futility [“a fundamental assertion about the intractability of the patient's disease or about the impotence of the doctor's treatment to alter the course” (Christakis, 1999, p. 205)] can legitimately justify a case for withdrawing life-sustaining (costly) treatment P-CA (Christakis, 1999; Luchetti, 2013) and therefore holds resource management implications. Medical professionals' withdrawal of treatment in cases of futility, is determined and underpinned by the professional principle and ethical priority of patient “best interest” (British Medical Association, 2007). However, the resource management connotations of prognostication can present professional dis-ease, challenging what Mechanic (2008) describes as the essential and fundamental norms of medical professionalism. In turn, physicians' resource management role might be interpreted to threaten aspects of medical professional integrity and identity, predicated on the ethical principle of “best interest” in the P-CA prognostication context. Within this milieu, we argue that prognosticators perform risk work by way of serving a dual role in relation to managing these risks to professional norms, integrity and identity, stemming from the potential managerialist connotations of prognostication. Where they indicate a prognosis of futility, and (costly) life-sustaining treatment is consequently withdrawn, prognosticators afford professionals the ability to meet managerialist demands and discharge managerial responsibilities, in a way consistent with their professional best interest principles. When treatment is withdrawn in the name of best interest owing to futility, the need for further (costly) treatment is negated, and without the requirement for medical professionals to deliberate on a more overtly financially informed decision, thus leaving their professional and ethical integrity and identity intact. Prognosticators can therefore be observed to mediate tension between hybrid managerial-professional imperatives, and in turn, risks to professional norms, integrity and identity. Further, since prognosticators provide an “objective” and “scientific” indication of likely futility, the decision to withdraw life-sustaining treatment on this basis is deemed professionally defensible, justifiable and ethically acceptable. Here, decision-making might be considered biologically and scientifically “preordained” or “fixed” (Christakis, 1999, p. 206) since, as identified in “Managing professional risk through devolving-dispersing-diluting-delegating”, it is devolved (and responsibility for it delegated) to prognosticators and science. Scientific and “objective” prognosticators in effect “speak for themselves,” objectifying decision-making and negating the need for a more subjective prognosis and decision-making surrounding the continuation of treatment. In turn, resource management might therefore be interpreted as a further example of professional delegation to prognosticators and science. It is through these mediating mechanisms that the risk work performed by prognosticators serves to manage the risk presented to professional norms, integrity and identity, by the P-CA prognostication process and its managerialist associations.

## Psychosocial risk and individual professionals – Affective and protective functions of prognosticators

In this final section, we argue for the affective and protective role that prognosticators play in managing professional psychosocial risk presented by the prognostication process, as a component of their risk work.

Demonstrating the relationship between the individual *professional* and collective *profession* (Kluge, 2007), prognostication is a practice undertaken by the medical profession on behalf of society, entailing collective professional risk. However, it is individual professionals who engage in and enact the process of prognostication and who are subject to these professional risks at an individual professional level. The “obligations of the profession” collectively translate to professional obligations individually (Kluge, 2007, p. 57), transferring professional risk in the process. Prognostication is difficult, uncertain, emotionally distressing, and highly clinically, bioethically and professionally consequential (Christakis, 1999). As such, medical professionals find prognostication concerning death, in particular, to be psychologically and intra-personally stressful and troubling (Christakis, 1999). Owing to the multitude of professional risks that it presents and the emotive context in which it is situated, we argue that prognostication P-CA carries with it a potentially significant psychosocial burden for individual medical professionals engaged in the process and practice - it presents *psychosocial* professional risk. By managing some of the initial professional risks that contribute to the psychosocial risk associated with prognostication however, through the ways of risk work described thus far, we argue that prognosticators simultaneously serve affective and protective professional functions; their use allays the professional impact of prognostication, and psychosocial risk as a consequence. In instances where an individual clinician “gets it wrong” for example, prognosticator use functions not only to manage professional risks to credibility, trust and power (by devolving-dispersing-diluting-delegating), but it also allays the psychosocial impact of “getting it wrong” on individual professionals [engendering, for example, potential guilt, anxiety, sense of moral burden, and responsibility (Christakis, 1999)] in the process. Thus, risk work performed by prognosticators includes an affective and professionally protective role, reducing psychosocial risk presented by the prognostication process P-CA.

## Discussion – A theoretical model, caveats and contributions

**Figure 1:**
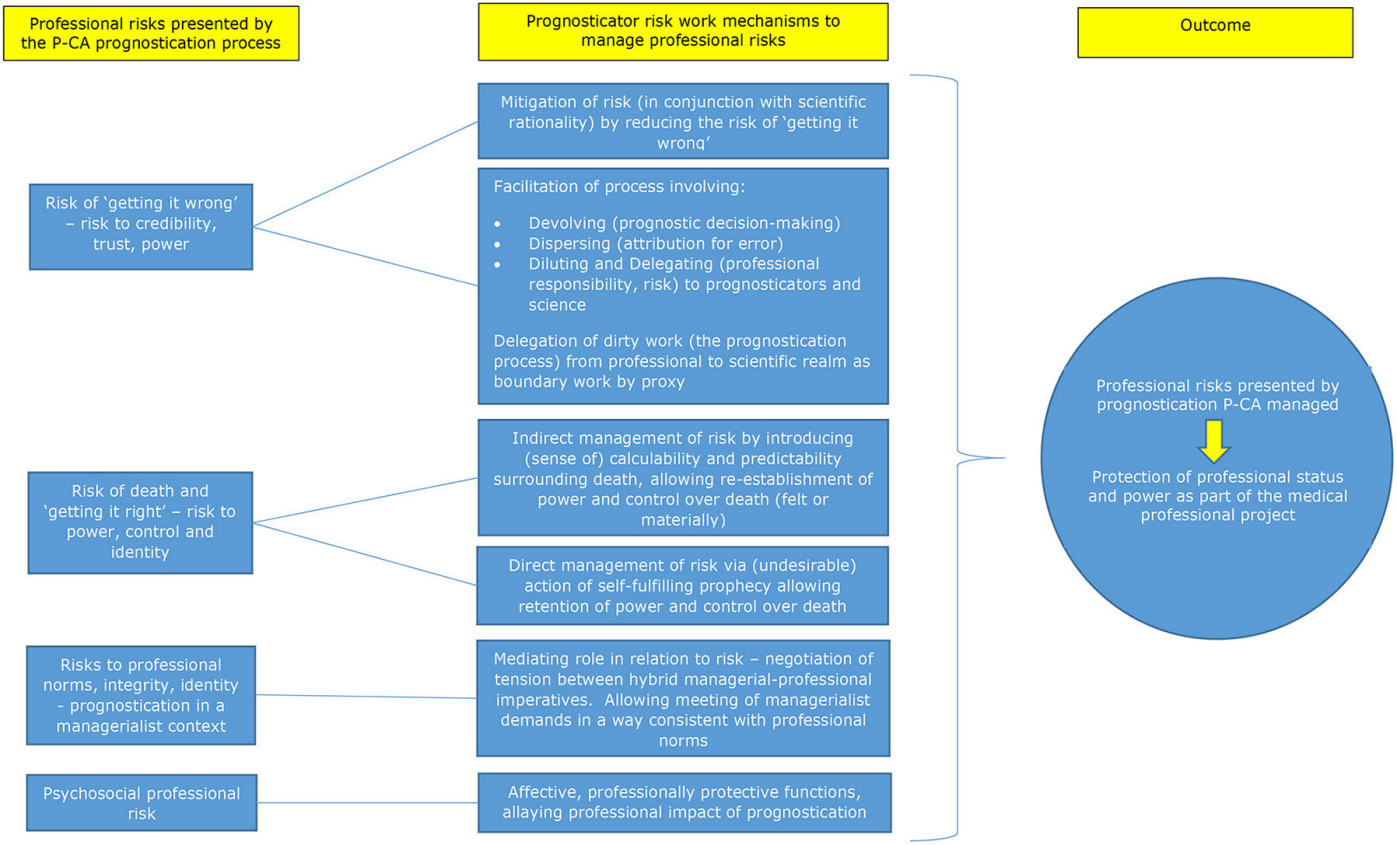
Theoretical model of the professional risk management functions of prognosticators in the context of prognostication post-cardiac arrest.

The prognostication process P-CA is challenging and suffused with clinical, bioethical and professional uncertainty and risk. Underpinned by sociological analyses of risk and uncertainty, and professions, we have argued that the medical profession's advocation for and use of prognosticators to guide the prognostication process, can be interpreted as a professional attempt to manage *professional* risk presented by the prognostication process P-CA. We have introduced the conceptualization of prognosticators as serving professional functions through the performance of “risk work,” mediating tension between the professional duty to prognosticate and professional risks presented. In explicating the (hitherto less enunciated) professional risk management functions of prognosticators, we have identified areas of professional risk presented by the P-CA prognostication process, the nature of risk work performed by prognosticators, how this functions to address professional risk and to what effect. Namely, we have identified the role that prognosticators and scientific rationality play in managing professional risks to professional credibility, trust and power, stemming from the risk of “getting it wrong.” Through performing risk work involving the *mitigation* of risk and the facilitation of a *devolving* (decision-making)-*dispersing* (attribution for error)-*diluting-delegating* (the locus of responsibility) process, prognosticator use serves to allay these professional risks (*dirty work*). We have illustrated how risks to professional power, control and identity presented by the requirement to confront death during prognostication, are managed directly and indirectly by the risk work of prognosticators. We detailed how this management is achieved through prognosticator use introducing (a sense of) *calculability* and *predictability*, and power and control over death *indirectly*, and also by affording professionals power and control over death more *directly* (and less desirably), through the action of a *self-fulfilling prophecy*. Further, we explained the *mediating* role that prognosticator risk work performs in managing risks to professional fundamental norms, integrity and identity, when considered in the context of managerialism. This mediating role affords professionals the ability to negotiate hybridity and meet managerialist demands in a way consistent with professional norms. Finally, we argued for an *affective* and *protective component* to prognosticator risk work, in relation to the management of individual psychosocial professional risk presented by the challenging, uncertain and highly consequential nature of the prognostication process. Here, by managing professional risks which contribute to the genesis of psychosocial risk and reducing the professional impact of the prognostication process, through their risk work, prognosticators simultaneously serve *affective* and *professionally protective* professional functions, managing psychosocial risk as a consequence. These arguments are summarized in Figure 1, which presents a theoretical model of the professional risk management functions of prognosticators in the context of prognostication P-CA.

### Caveats and opportunities

We caveat our argument and analysis, firstly by acknowledging their theoretical nature *a priori*. Empirical work exploring prognostication P-CA as a situated practice might utilize these theoretical ideas and conceptual arguments as a framework to explore the nature of professional risk and the risk work role of prognosticators, in the context of their manifestation and enactment in the “everyday” reality of clinical practice. In this way, the arguments presented may be further developed, refined and augmented. Secondly, our aim in this paper was to provide a theoretical analysis and interpretation of the professional functions of prognosticators in relation to the management of risk and uncertainty presented by the P-CA prognostication process. We acknowledge that prognostication P-CA is inherently complex and that the view we have presented here, necessarily for analytical purposes, constitutes a simplification of the process, its variables and implications. Although we have attempted to incorporate consideration of different eventualities, possibilities and outcomes in the development of arguments, we do not intend to deny this inherent complexity and suggest that studies of applicability and difference across situational contexts, constitute a fruitful area for further theoretical and empirical work.

### Contributions to knowledge

The analysis presented has drawn upon extant sociological theory, applying and extending it to the context of the medical profession's practice of prognostication P-CA. In doing so, this paper contributes to sociological theorizing surrounding risk and uncertainty, and professions. Through our critique of the process and practice of prognostication more broadly, we contribute to and inform “an emerging sociology of prognosis” (Timmermans and Strivers, 2018, p. 13).

With regard to the sociology of professions, firstly, we contribute to theorizing the hitherto less enunciated *professional* functions of prognosticators in relation to the management of risk and uncertainty, and locate this within a specific (P-CA) context. As a component of risk work performed by prognosticators P-CA, we also introduce the notion of “boundary work by proxy” as the performance of boundary work by prognosticators, on behalf of medical professionals. Further, we identify its nature and purpose in delegating the dirty work of professional risk and responsibility associated with the prognostication process, to outside of the professional and toward the (inexact) scientific realm, to maintain professional credibility, trust and power, consistent with the medical profession's professional project. In doing so, we demonstrate how “dirty work” functions to defend and maintain facets of professional status, as part of the medical profession's professional project, which has tended to be overlooked in prior conceptualizations of dirty work (Miner, 2019). Furthermore, we identify the potential for medical professional prognoses of death to operate as a self-fulfilling prophecy, highlighting an instance in which power and its exercise can be analyzed as professionally problematic and undesirable in the context of the professions. Finally, we contribute to understanding surrounding the nature, practice and social organization of professional risk work, potentially as an aspect and/or consequence of “new professionalism” in the context of managerially-driven healthcare.

Gale et al. (2016, p. 1046, 1065) identify a series of neglected areas of knowledge surrounding risk work, to which we contribute theoretically. We have considered, for example, the “impact of risk on the nature…of healthcare work” using risk work to “develop our understanding of these practices.” We have considered what risk work is *doing* in the context of a broader sociological framework, and have incorporated consideration of the issues of professional credibility and identity in the context of risk work. Further, in exploring *professional* risk work, we have identified a specific *type* of risk work (along with discrete functions), contributing to understanding surrounding the impact of risk work on the professions, how professional identity is mediated in the context of risk, and in particular, micro (prognostication P-CA) and macro (managerialist, healthcare) risk contexts. Indeed, to our knowledge, this is the first analysis of risk work applied to the context of distinctly *professional* risks and its management broadly, and particularly, within medical professional, prognostication, and P-CA contexts.

## Conclusion

Collectively, the arguments presented in this paper demonstrate the risk work role that prognosticators perform in managing professional risk - risk to central tenants of medical professional status, presented by the prognostication process P-CA. As such, we conceive of the use of prognosticators as a professional response - a technical solution to the professional problem of risk inherent within the P-CA prognostication process, employed as part of the medical professions' engagement in their ongoing professional project to gain and maintain professional status and power. Whilst prognosticators have as yet failed to deliver certainty in relation to prognostication P-CA as their primary purpose, when considered within a wider socio-cultural context, their use can be observed to serve other important *professional* functions relating to the management of risk and uncertainty presented by the prognostication process, particularly in the P-CA context.

## Data availability statement

The original contributions presented in the study are included in the article/supplementary material, further inquiries can be directed to the corresponding author/s.

## Author contributions

SF-R wrote the first and subsequent drafts of the manuscript. Both authors contributed to the conception, development of the manuscript content, revision, read, and approved the submitted version.

## Funding

This work was funded by a small grant awarded to ST from the School of Nursing, University of Nottingham. Open access publication fees received from University of Nottingham.

## Conflict of interest

The authors declare that the research was conducted in the absence of any commercial or financial relationships that could be construed as a potential conflict of interest.

## Publisher's note

All claims expressed in this article are solely those of the authors and do not necessarily represent those of their affiliated organizations, or those of the publisher, the editors and the reviewers. Any product that may be evaluated in this article, or claim that may be made by its manufacturer, is not guaranteed or endorsed by the publisher.
